# Evaluation of hybrid stroke quality indicators by integrating NIHSS and claims data for improved outcome prediction

**DOI:** 10.1038/s41598-025-25979-1

**Published:** 2025-11-07

**Authors:** Thomas Datzmann, Caroline Lang, Falko Tesch, Melissa Spoden, Patrik Dröge, Franz Ehm, Ekkehard Schuler, Christos Krogias, Christian Günster, Jochen Schmitt, Christoph Gumbinger, Jessica Barlinn

**Affiliations:** 1 Center for Evidence-Based Healthcare, Medical Faculty and University Hospital Carl Gustav Carus, TUD Dresden University of Technology, Dresden, Germany; 2 Wissenschaftliches Institut der AOK (AOK Research Institute), Berlin, Germany; 3 Helios Kliniken GmbH, Berlin, Germany; 4 Department of Neurology, Evangelisches Krankenhaus Herne, Academic Teaching Hospital of the Ruhr University Bochum, Herne, Germany; 5 Department of Neurology, Heidelberg University Hospital, Heidelberg, Germany; 6 Department of Neurology, University Hospital and Faculty of Medicine Carl Gustav Carus, Technische Universität Dresden, Dresden, Germany

**Keywords:** Stroke, Health care quality assurance, Routinely collected health data, Health care quality indicators, Health care, Medical research, Neurology

## Abstract

Accurately measuring the quality of stroke care based on claims data alone is challenging. Traditional outcome metrics, e.g. mortality rates, do not capture the effectiveness of critical stroke care processes. We aimed to develop hybrid quality indicators (QIs) by integrating clinical stroke severity data with claims data. Claims data were linked to patient-level clinical data from 15 hospitals (2017–2020) and harmonized in the Observational Medical Outcome Partnership (OMOP) data model. Inclusion criteria, outcomes and risk factors were developed by medical expert panels. We applied machine learning for modeling the outcomes *30-day-mortality*, *reinfarction within 90 days*, and *care degree increase within 180 days*. We compared extreme gradient boosting (XGBoost) models with and without the National Institutes of Health Stroke Scale (NIHSS) using Receiver-Operating-Characteristic-Area-Under-the-Curve (ROC-AUC) and Brier Score (BS). Hospitals were ranked according to the impact of each QI using Standardized Mortality Ratios (SMRs). The study included 9,348 ischemic (I63) and 1,554 hemorrhagic (I61) strokes, with NIHSS available for 5,012 patients. For all three outcomes, disease severity as measured by NIHSS was the most important determinant. The predictive power of the hybrid models was higher than that of models based on claims data alone. For SMR, the influence of NIHSS was greater than that of age, the most important variable in the claims data model. The results were consistent between the two entities, different outcomes, and sensitivity analyses. Including NIHSS information alongside claims data improves the risk adjustment of quality indicators.

**Supplementary Information:**

The online version contains supplementary material available at 10.1038/s41598-025-25979-1.

## Introduction

Stroke is one of the leading causes of disability and mortality worldwide, placing a significant burden on healthcare systems and requiring high quality care to optimize patient outcomes^1^. However, measuring the quality of stroke care is challenging, especially when relying on claims data alone. Claims data, which are collected for administrative rather than clinical purposes, often lack the details needed to fully capture the complexity of stroke care quality^2^. These data are generally limited to basic information on diagnosis, treatment, and hospital stay, which may overlook critical aspects of patient management and specific clinical information that are essential for accurate quality assessment. In particular, outcomes and risk factors are limited^3–7^, so that residual confounding and the associated potential lack of validity of existing quality indicators (QIs) cannot be ruled out.

In Germany, external quality assurance for stroke care is only mandatory for institutions certified by the National Stroke Association, which requires certified stroke units to systematically collect structured clinical data^8^. However, these data are often collected separately and are not always readily accessible for data linkage, posing a challenge for comprehensive quality assessment and analysis in big data sets.

Traditional metrics, such as mortality rates and length of hospital stay, provide only a limited view of quality of care and may not reflect the effectiveness of critical processes, such as timely reperfusion therapy or comprehensive therapeutic treatments such as physiotherapy and speech therapy. Therefore, there is a need for hybrid QIs that can combine both claims data and additional clinical information to provide a more comprehensive view of the quality of stroke care.

In this project, we took the first step toward linking clinical information directly out of data warehouses of the individual participating clinics with data from statutory health insurers outside of study data, not directly collected and documented for the study purpose. In addition to feasibility, the aim was to expand the quality assessment procedure used in Germany (QSR - Qualitätssicherung mit Routinedaten), which exclusively uses data from statutory health insurers to compare the quality of hospitals, to include clinical parameters so that an “extended” QSR procedure could also be carried out for severity-sensitive stroke. The emphasis was on a minimally expanded model. We developed hybrid QIs including clinical information on stroke severity based on the National Institutes of Health Stroke Scale (NIHSS) at hospital admission.

## Results

### Study population

A total of 1,554 patients with a main discharge diagnosis of I61 and 9,348 with I63 (ICD-10-GM) were recorded in the claims data alone during the study period from 2017 to 2020 (Table  2). Nine out of 15 hospitals provided information on the NIHSS score at admission. A total of 511 patients were included with I61 and 4,501 with I63 for the hybrid data (Table  2). Patient characteristics were reported separately for those with and without NIHSS scores for both entities I61 and I63 (Table  1). In addition to the NIHSS status, percentages of socio-demographic factors (age, sex), comorbidities, medications in the two years prior to the index stroke and other patient characteristics were shown. There were no major differences in these between the population with and without NIHSS information. The ratio between the number of patients with and without event was moderate for all three binary outcomes examined. The outcome *reinfarction within 90 days* was represented by an almost equal rate (1:1), while the ratio for *care degree increase within 180 days* and *30-day mortality* increased to 1:7 (event: no event). Patients with I63 reached the outcome *30-day mortality* less frequently than patients with I61 (Table  2).

**Table 1:** Baseline information on patient characteristics and risk factors for I61 and I63 are shown, stratified by the availability of the NIHSS. mRS: modified Rankin scale at hospital discharge (physician-reported disability scale).

	I61				I63			
	Without NIHSS	%	With NIHSS	%	Without NIHSS	%	With NIHSS	%
Total N	1043	100	511	100	4847	100	4501	100
Median age at index stroke [range]	78.3 [24–102]	–	78.9 [25–97]	–	78.6 [19–105]	–	78.0 [19–102]	–
Sex (female)	548	52.5	250	48.9	2605	53.7	2220	49.3
Care degree								
None	596	57.1	312	61.1	2957	61.0	2912	64.7
Degree 1	36	3.5	14	2.7	141	2.9	145	3.2
Degree 2	157	15.1	64	12.5	762	15.7	649	14.4
Degree 3	145	13.9	61	11.9	572	11.8	453	10.1
Degree 4	83	8.0	43	8.4	329	6.8	268	6.0
Degree 5	26	2.5	17	3.3	86	1.8	74	1.6
Thrombectomy in index stay	–	–	–	–	494	10.2	481	10.7
Predictors (scored 2 years before, including index)								
Thrombocytopenia	18	1.7	2	0.4	47	1.0	48	1.1
STEMI (ST-elevation myocardial infarction)	56	5.4	39	7.6	328	6.8	308	6.8
Diabetes	226	21.7	138	27.0	1151	23.7	1076	23.9
PSD (post stroke depression)	110	10.5	59	11.5	458	9.4	401	8.9
HYPERCHOL (hyperholesterinaemia)	552	52.9	316	61.8	3193	65.9	3372	74.9
POAD (peripheral arterial occlusive disease)	116	11.1	54	10.6	661	13.6	573	12.7
NICOTINE (nicotinabus)	122	11.7	60	11.7	828	17.1	775	17.2
ERYTHROP (erythrocytopenia)	13	1.2	8	1.6	32	0.7	36	0.8
CRHINO (chronic rhinosinusitis)	41	3.9	14	2.7	171	3.5	170	3.8
DEMENTIA	212	20.3	117	22.9	900	18.6	777	17.3
OSS (obstructive sleep apnoea syndrome)	36	3.5	25	4.9	214	4.4	211	4.7
Iron deficiency anaemia after blood loss	12	1.2	7	1.4	70	1.4	79	1.8
Other iron deficiency anaemias	28	2.7	8	1.6	122	2.5	117	2.6
Pulmonary embolism	19	1.8	7	1.4	35	0.7	28	0.6
Nutritional deficiency	59	5.7	23	4.5	260	5.4	185	4.1
Atrial fibrillation and flutter	222	21.3	121	23.7	941	19.4	770	17.1
Hypertension	123	11.8	64	12.5	673	13.9	549	12.2
Coagulopathies	67	6.4	27	5.3	196	4.0	192	4.3
OESTROGEN (estrogen therapy)	43	4.1	17	3.3	152	3.1	154	3.4
Cardial implantation	78	7.5	38	7.4	450	9.3	379	8.4
Hypertensive kidney disease with renal insufficiency	13	1.2	5	1.0	60	1.2	53	1.2
Renal insufficiency	271	26.0	130	25.4	1389	28.7	1026	22.8
Dialysis	35	3.4	35	6.8	35	0.7	35	0.8
STENOSIS (cerebral atherosclerosis)	61	5.8	46	9.0	420	8.7	378	8.4
Medications (scored 2 years before index)								
ARBP (angiotensin receptor blocker pure)	197	18.9	102	20.0	1032	21.3	849	18.9
ARBC (angiotensin receptor blocker combined)	134	12.8	67	13.1	718	14.8	603	13.4
STATINS (statins)	368	35.3	209	40.9	1801	37.2	1626	36.1
ANTICOA (anticoagulants)	503	48.2	266	52.1	2246	46.3	1967	43.7
ANTIPSY (antipsychotics)	121	11.6	65	12.7	540	11.1	502	11.2
BETABP (beta blocker pure)	535	51.3	293	57.3	2695	55.6	2348	52.2
BETABC (beta blocker combined)	14	1.3	15	2.9	115	2.4	139	3.1
Median length of stay in days [range]	10.0 [0.13–94.7]		10.0 [0.16–83.5]		7.8 [0.13–85.1]		7.7 [0.14–71.8]	
Clinical parameters								
NIHSS (on admission)								
Total N			511	100	–	–	4501	100
0–5 points	–	–	169	33.1	–	–	2569	57.1
6–22 points	–	–	282	55.2	–	–	1667	37.0
>22 points	–	–	60	11.7	–	–	265	5.9
mRS (at discharge)								
Total N			436	100			4188	100
0–2 points	–	–	158	36.2	–	–	2484	59.3
3–5 points	–	–	206	47.2	–	–	1438	34.3
6 points	–	–	72	16.5	–	–	266	6.4

**Table 2:** Case numbers for claims and hybrid data including three binary outcomes (*30-day mortality*, *reinfarction within 90 days*, and *care degree increase within 180 days*) and their class ratios are shown for I61 and I63, respectively. The outcome reinfarction within 90 days counts all hospital admissions with a main discharge diagnosis of stroke (I61/I63) within 90 days of the index stroke. Both I-codes count as a re-event regardless of the type of the index stroke (I61 or I63).

Outcome	Status	I61				I63			
		ClaimsData	Class imbalance	Hybrid data	Class imbalance	ClaimsData	Class imbalance	Hybrid data	Class imbalance
		N		N		N		N	
30-day mortality	Alive	1080	~ 1:2	388	~ 1:3	8159	~ 1:7	3969	~ 1:7
	Dead	474		123		1189		532	
	Total	1554		511		9348		4501	
Reinfarction within 90 days	No_reinfarct	422	~ 1:1	173	~ 1:1	4854	~ 1:1	2375	~ 1:1
	Reinfarct	663		223		3319		1612	
	Total	1085		396		8173		3987	
Care degree increase within 180 days	No_increase	742	~ 1:3	284	~ 1:4	6413	~ 1:5	3149	~ 1:5
	Increase	232		76		1368		639	
	Total	974		360		7781		3788	

### Medical expert panels

Medical expert panels identified a total of 35 potential risk factors for I61 and 39 potential risk factors for I63 based on claims data. Selected risk factors included age, sex, previous ST-segment elevation myocardial infarction (STEMI), existing coronary artery bypass, use of anticoagulants and other selected medications, thrombectomy during the index hospitalization (relevant only for I63), and selected Elixhauser comorbidities^9,10^ were selected as risk factors. A full list is available as a supplementary file (Table ~ S1). Within the clinical data, we used the physician-reported NIHSS score as a risk factor and as a proxy for stroke severity.

### Goodness of fit measures

In order to discern the influence on modeling by integrating the NIHSS, performance measures were compared for the XGBoost models trained with and without this clinical parameter, keeping all other aspects of model design constant. The goodness of fit measures were always higher for models fit on the hybrid data (Table  3). The influence of NIHSS on model performance was particularly pronounced for *30-day mortality*. The inclusion of the NIHSS increased the ROC-AUC values by up to 15% in absolute terms.

For the outcome *30-day mortality*, a sensitivity analysis was performed excluding patients with a NIHSS of 32, as these patients are more likely to require mechanical ventilation. Mechanical ventilation makes it difficult to assess stroke severity and hints at other factors potentially influencing readmission or mortality. Sensitivity analysis showed that excluding these patients had no effect on model performance (Table  3).

**Table 3:** XGBoost test data performance for comparison between analyses based on claims or hybrid datasets. Results are shown for three outcomes and for the sensitivity analysis (based on *30-day mortality* only; bold). The analysis based on hybrid data (including of NIHSS) always shows a better goodness of fit (higher ROC-AUC, lower Brier Score). The effect is most pronounced for the first outcome, *30-day mortality*, for both entities.

Model: XGBoost		I61		I63		Model: XGBoost sensitivity		I61		I63	
Outcome	Metric	ClaimsData	HybridData	ClaimsData	HybridData	Outcome	Metric	ClaimsData	HybridData	ClaimsData	HybridData
30-day mortality	ROC-AUC	0.6482	0.8002	0.7579	0.8736	**30-day mortality**	**ROC-AUC**	**0.6409**	**0.8136**	**0.7577**	**0.8798**
	Sens	0.9258	0.9524	0.9881	0.9747		**Sens**	**0.9525**	**0.9464**	**0.9930**	**0.9784**
	Spec	0.1353	0.3226	0.0802	0.3571		**Spec**	**0.1176**	**0.2647**	**0.0482**	**0.3066**
	Accuracy	0.6885	0.8088	0.8746	0.9037		**Accuracy**	**0.7013**	**0.7877**	**0.8789**	**0.9075**
	Kappa	0.0762	0.3361	0.1066	0.4122		**Kappa**	**0.0900**	**0.2621**	**0.0673**	**0.3679**
	PR-AUC	0.8131	0.9276	0.9526	0.9778		**PR-AUC**	**0.8008**	**0.9279**	**0.9546**	**0.9803**
	Precision	0.7139	0.8264	0.8826	0.9211		**Precision**	**0.7150**	**0.8092**	**0.8837**	**0.9228**
	F	0.8062	0.8850	0.9324	0.9471		**F**	**0.8168**	**0.8724**	**0.9351**	**0.9498**
	Brier_Score	0.3115	0.1912	0.1254	0.0963		**Brier_Score**	**0.2987**	**0.2123**	**0.1211**	**0.0925**
Reinfarction within 90 days	ROC-AUC	0.5586	0.6944	0.6419	0.6733	**Care degree increase within 180 days**	**ROC-AUC**	**0.8538**	**0.8734**	**0.8824**	**0.9137**
	Sens	0.1721	0.4898	0.8695	0.8420		**Sens**	**0.9217**	**0.9367**	**0.9369**	**0.9595**
	Spec	0.9063	0.7619	0.2859	0.3920		**Spec**	**0.5714**	**0.5263**	**0.4701**	**0.4681**
	Accuracy	0.6210	0.6429	0.6325	0.6610		**Accuracy**	**0.8429**	**0.8571**	**0.8560**	**0.8776**
	Kappa	0.0895	0.2575	0.1693	0.2493		**Kappa**	**0.5225**	**0.5033**	**0.4475**	**0.4926**
	PR-AUC	0.4723	0.5949	0.6959	0.7073		**PR-AUC**	**0.9325**	**0.9271**	**0.9685**	**0.9795**
	Precision	0.5385	0.6154	0.6404	0.6731		**Precision**	**0.8811**	**0.8916**	**0.8940**	**0.9001**
	F	0.2609	0.5455	0.7376	0.7481		**F**	**0.9009**	**0.9136**	**0.9149**	**0.9289**
	Brier_Score	0.3790	0.3571	0.3675	0.3390		**Brier_Score**	**0.1571**	**0.1429**	**0.1440**	**0.1224**

### Variable importance measures

NIHSS was ranked as the most important parameter by the models for the outcomes *of 30-day mortality* and *reinfarction within 90 days*. NIHSS was at least in second place for the outcome *care degree increase within 180 days* (see plots to the right of Figure ~ S1&S2 in the Supplementary Appendix). These results were consistent across both entities I61 and I63. The NIHSS also remained the most prominent parameter in the sensitivity analysis (Figure ~ S3). We also had information on the modified Ranking Scale (mRS) on discharge. However, this score was highly correlated with the NIHSS at admission, so we focused exclusively on the NIHSS. In addition, we would have lost further patients by using the mRS, as the coverage here was even lower than for the NIHSS. The possible variability of disease severity is also better represented in the NIHSS (43 versus 7 categories). Other potentially important parameters from the clinical information systems were not available to us. Among other things, further information on the socio-demographics of the patients (income, education, marital status) was lacking. Important physical characteristics (weight, height, waist-to-hip ratio) were also inaccessible. Process indicators (Event-to-Door or Door-to-Needle times) were also completely missing. Although, no laboratory values or physiological findings (e.g. blood pressure) could be included. Within the models based exclusively on claims data, age and the degree of care needed before hospital admission had a certain influence. In the case of ischemic stroke, the execution of a thrombectomy was also influential. The influence of the other used claims-based variables was negligible for the modeling (Figure ~ S1-S3 and Table ~ S1).

### Standardized event ratios

**Fig. 1:**
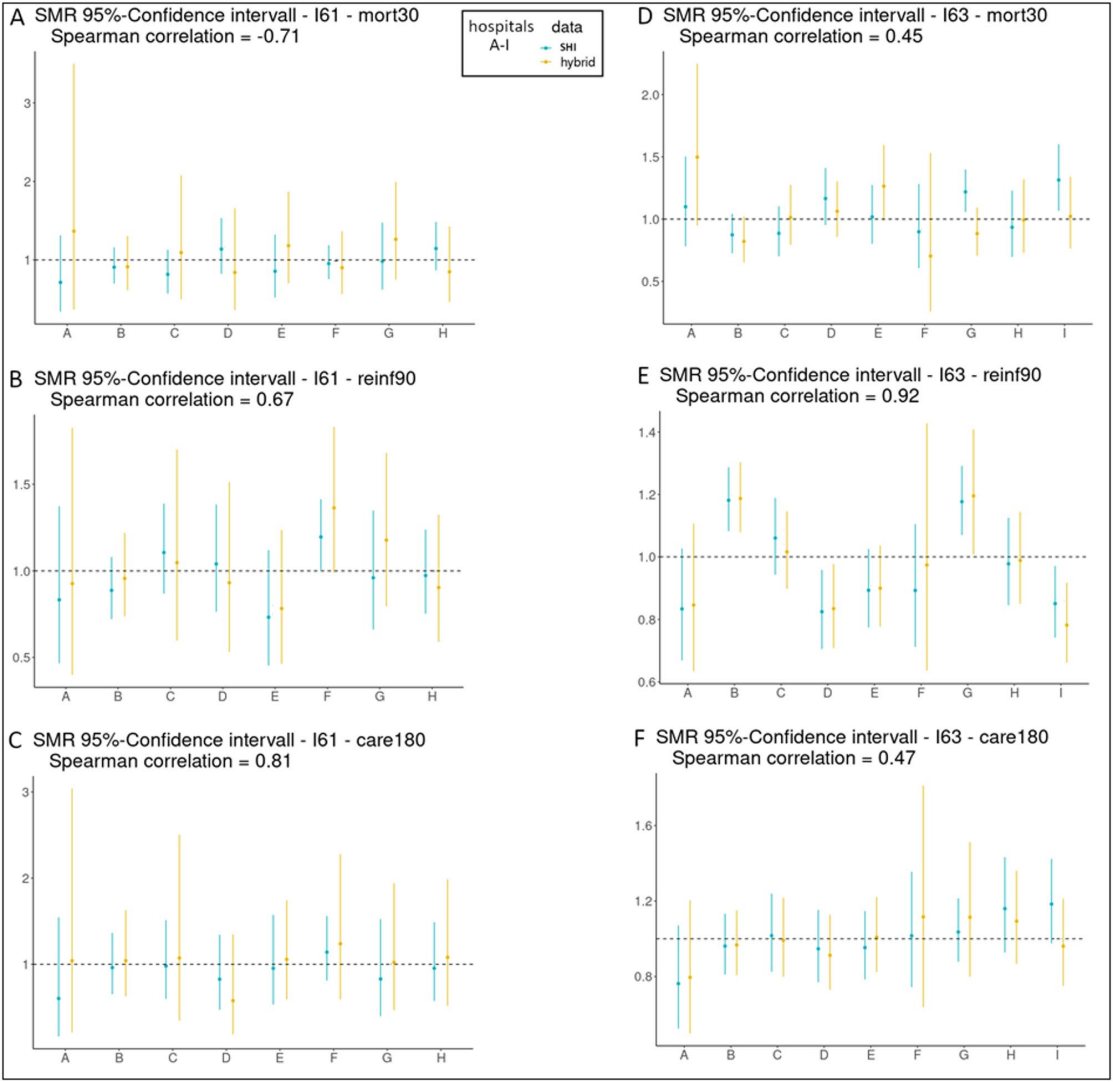
Comparison of hospital ranking based on SER/SMR (XGBoost model) between claims data and hybrid data. Data are shown for three outcomes are shown: *30-day mortality*, *reinfarction within 90 days*, and *care degree increase within 180 days*; (**A**–**C**) data for I61; (**D**–**F**) data for I63; SMR (Standardized Mortality Ratio) with 95% confidence interval and Spearman correlation between claims data and hybrid data are given for each comparison.

We compared the ranking of hospitals according to their Standardized Event Ratios (SER) between the XGBoost model with (hybrid data) and without NIHSS information (claims data) for all three outcomes (Fig. 1). The SER differed between the analysis of claims- and hybrid data for all comparisons with a Spearman rank correlation between − 0.71 and 0.92, depending on the outcomeand entity (I61 or I63). In particular, the standardized mortality rate (SMR - based on *30-day mortality*) showed mostly poor rank correlations (rho’s: -0.71, 0.45). The same weak correlations were found in the sensitivity analysis (Figure ~ S4).

## Discussion

Our results are consistent between patients with ischemic and patients with hemorrhagic stroke (I63 or I61) and provide evidence for improved risk adjustment by adding clinical information on the NIHSS to quality indicators based on claims data. The inclusion of the NIHSS as a proxy for stroke severity significantly improved model performance for key outcomes, particularly for *30-day mortality.* The observed improvements, including an absolute increase in ROC-AUC up to 15%, underscore the value of clinical severity data in improving prediction accuracy, particularly for outcomes that are highly sensitive to patient status at admission. This finding supports the potential utility of hybrid models combining claims data with clinical information on NIHSS in quality assessment, although such improvements depend on the consistent availability of clinical data. In our analysis, the limited availability of NIHSS data across hospitals (9 out of 15) and the reduced sample size for the hybrid dataset (only 5,012 patients in total) highlight a key challenge in developing hybrid QIs. Although most hospitals probably collected the clinical data, they were not available for data linkage due to a lack of interoperability between different systems for the medical documentation and undergoing maintenance. It was not possible to determine whether the failure was due to chance or induced selection bias in our sample. This limitation in data access may limit generalizability to broader hospital populations and regions. However, despite the reduced sample size, there were no major differences in baseline characteristics between patients with and without NIHSS information, suggesting that the subset may still be representative. Other studies^11,12^ also reported problems getting NIHSS information complete for their study population. The loss of patients was of the same order of magnitude as ours. Numerous studies^11–16^ have already shown that adding the NIHSS leads to an improvement in risk adjustment for stroke patients. Particularly for outcomes that depend on the patient’s condition on admission, such as *30-day mortality*, these studies^12,14,16^ suggest that the addition of stroke severity (NIHSS as the most commonly used international parameter) is essential for risk adjustment. Evidence for the influence of physiological parameters and laboratory values (e.g. blood markers) on risk modeling is weak^6^.

Applied QIs based on claims data often do not take into account the complex nature of stroke care, particularly in the acute inpatient setting, where effective treatment requires not only prompt intervention but also coordinated care involving different healthcare professionals and specialized protocols^17,18^. This is especially true as they do not include clinical parameters that have a major impact on outcome.

The NIHSS is widely used in clinical and research settings to determine stroke severity, guide treatment decisions, and predict patient outcomes^19,20^. In stroke care, the NIHSS serves as a critical indicator of patient´s condition at admission, helping to ensure that comparisons of outcomes between hospitals or treatments take into account differences in initial stroke severity. This is particularly important in research context such as interventional studies. The high ranking of the NIHSS as the most important variable for predicting *30-day mortality* and *90-day reinfarction* in our study is consistent with its clinical role as an indicator of stroke severity. The robustness of the NIHSS as a predictor, even after excluding severely affected patients (NIHSS = 32) in the sensitivity analysis, strengthens its validity as a quality indicator. This finding suggests that the NIHSS remains a critical factor across different model configurations, and it may serve as a stable component in hybrid quality assessments for German hospitals.

Quality in health care has attracted increasing public interest, especially in recent years^17^. Publicly accessible quality reports and rankings for physicians and hospitals are now widely available. Moreover, transparent reporting on the quality of health care has become a central focus of recent health policy reforms. In this context, the provision of reliable information based on solid and sound methods is crucial. As our data show, the addition of clinical information in a meaningful way has led to changes in quality assessment metrics. Significant differences in hospital rankings based on standardized event rates (SERs) between models with and without the NIHSS implicate that using claims data alone may misrepresent hospital performance. The low correlations observed for *30-day mortality* SERs, particularly for haemorrhagic stroke, suggest that the inclusion of NIHSS significantly shifts quality assessment metrics. These shifts underscore the potential risk of misclassification in hospital rankings when severity data is excluded, with potential implications for accountability and quality improvement efforts in stroke care. Our analyses show that mortality alone (without the use of additional information on stroke severity) should not be used as quality indicator based on claims data. This is particularly important given the different levels of stroke care - from specialized centers to telemedicine network hospitals - where accurate assessment is essential to ensure that quality standards are consistently met^21^.

The integration of clinical severity data, such as the NIHSS, into claims data-based models provides a methodological advantage, but also presents practical challenges. Hybrid QIs require robust clinical data collection systems and close cooperation at several stages across institutions. While this study demonstrates that the addition of the NIHSS improves predictive power, the practical implementation of such hybrid indicators in real-world settings may require policy adjustments to support consistent, quality-driven data collection. Although, structured clinical data are collected between stroke units, they are not always directly accessible for extended use and big data analysis.

The observed high rate of reinfarction for haemorrhagic stroke (I61) is not entirely convincing. This is because hospital admissions coded as either I61 or I63 were both classified as reinfarction. It remains unclear whether misclassification of complications of the initial stroke as I61 or I63 may have contributed to the increased rate. In addition, sample bias for I61 cannot be excluded as a potential factor.

Given the demonstrated impact of the NIHSS on model accuracy, future work should explore expanding hybrid indicators to include additional clinical data where feasible, possibly integrating digital health tools to streamline data collection. Based on our findings, it is not possible to determine whether the addition of NIHSS in stroke provides sufficient risk adjustment or whether additional clinical parameters are needed. It may be helpful if hospitals were legally required to provide data on stroke severity for every stroke patient to improve comparability of quality of care between hospitals.

Although standardized data collection protocols exist in certified stroke units, there is a need for policy makers and healthcare providers to ensure comprehensive and comparable quality assessments. The need for cross-institutional consistency of data, data completeness and availability of data remains a critical factor for the reliable implementation of hybrid QIs in stroke care assessment.

Hybrid QIs may also have important economic and preventive implications. More precise, risk adjustment can reduce misclassification of hospital performance and enable comparisons between hospitals. As a consequence, more efficient allocation of healthcare resources could be guided and therefore support quality improvement. Ultimately, reduction of preventable complications and rehospitalization contributes to cost efficiency. Moreover, stroke prevention could be strengthened when systematic comparisons of risk-adjusted outcomes between hospitals are enabled. In this way, hybrid QIs have the potential not only to improve the accuracy of acute care evaluation but also to inform preventive strategies and foster more integrated models of stroke care.

## Methods

### Study design

Retrospectively, longitudinal claims data of the ”AOK – Die Gesundheitskasse” were linked to clinical data from 15 participating German hospitals on a per-patient basis (hybrid dataset) and harmonized in the Observational Medical Outcome Partnership (OMOP) data model^22^. The database and the data linkage and harmonization process have been described in more detail elsewhere^23,24^. Briefly, case definitions in the claims data were based on the respective coding systems for diagnoses (ICD-10-GM), the German modification of the International Classification of Procedures in Medicine (OPS), and prescriptions (Anatomical Therapeutic Chemical Code (ATC)). Inclusion criteria were hospital admissions with a primary diagnosis of stroke (hemorrhagic I61 or ischemic I63, ICD-10-GM), age ≥ 18 years. No further exclusions were defined. Several iterative medical expert panels were conducted to reach a consensus on inclusion criteria, relevant risk factors, and outcomes to be measured. The claims data included patient-related factors from sociodemography and comorbidities. Only factors that cannot be influenced by the hospital itself were included. However, process indicators (e.g., treatments) and outcome indicators were not selected as risk factors, as the quality indicator is intended to adjust for/detect potential quality differences in these areas.

### Outcomes

Three binary outcomes were considered: *30-day mortality*, *reinfarction within 90 days*, and *care degree increase within 180 days*. The *30-day mortality* was considered to have occured if there were up to 30 days between the date of admission and death. *Reinfarction within 90 days* was achieved if there were up to 90 days between the date of admission and a new hospital admission with a main discharge diagnosis of stroke (ICD 10: I61/I63). An *increase in the degree of care within 180 days* was defined as up to 180 days between the date of admission and an increase in the degree of care. In Germany, the degree of care is divided by five levels. A new classification or upgrade of the level within the time period was counted as an increase.

### Data preparation and modeling

Data preparation and cleaning have been described elsewhere in more detail^24^. Model building and comparative analyses were carried out using R 4.2.2^25^, mainly employing the *caret*^26^ package. We deployed Extreme Gradient Boosting (XGBoost)^27^ to estimate the probabilities of reaching the outcome for all patients. XGBoost is a widely used, powerful machine learning method, which has shown competitive performance in predicting stroke-related outcomes^28,29^. We applied a 70:30 train/test split and compared model performance with and without clinical information, always on the unseen test dataset. Among others, we relied on the Receiver-Operating-Characteristic-Area-Under-the-Curve (ROC-AUC), and the Brier Score (BS) as measures of comparison. Feature importance plots served to derive the effects of individual variables such as the National Institutes of Health Stroke Scale (NIHSS) at admission. The NIHSS was used as a surrogate for the severity of each patient’s stroke and was extracted from the clinical information systems of the included hospitals, where available. This widely used physician-reported score is a standardized clinical tool used to assess the severity of neurological impairment in patients who have suffered a stroke. The scale assesses key functions such as consciousness, vision, motor skills, speech, and sensory response, and provides a score that reflects the extent of the stroke. Scores range from 0 to 42, with higher scores indicating more severe impairment.

### Calculation of standardized event ratios (SER/SMR)

We used the *ems*^30^ package in R for indirect standardization of the event rates. To form the SER/SMR, the observed outcome event rates were set in relation to the expected event rates (probabilities calculated from the model). Confidence intervals (CIs) were generated using the Byar approximation. Finally, the SER was used to rank the hospitals in the study. The ranking based on claims data alone was compared with the ranking including NIHSS. The Spearman rank correlation was used as the statistical measure.

### Sensitivity analysis

As a sensitivity analysis, we repeated the entire process, but only for the primary outcome of *30-day mortality*. In addition, all patients with an NIHSS of 32 were excluded.

## Supplementary Information

Below is the link to the electronic supplementary material.

Supplementary Material 1

## Data Availability

The datasets generated and analysed during the current study are not publicly available due restrictions on the protection of personal data by German social law but access to the data can be granted under the circumstances defined in German social law (SGB V § 287). For information and assistance in these cases, **please contact the corresponding author Thomas.Datzmann@ukdd.de**.

## Declarations

### Adherence to guidelines and regulations

The study was carried out according to the Declaration of Helsinki and the recommendations of Good Practice Secondary Data Analysis (GPS), Good Practice Data Linkage (GPD) and Good Epidemiological Practice (GEP). Data linkage was carried out by the Trusted Third Party Office of the University Hospital Carl Gustav Carus and was based on approved § 75 applications (SGB X, German social law) from the responsible regulatory authorities. Individual patient consent was not required for the analysis and linkage of social data due to the approval of the § 75 applications by the respective regulatory authorities.

## References

[1] Martin, S. S. et al. 2024 Heart disease and stroke statistics: A report of US and global data from the American Heart Association. *Circulation*. **149**, e347–e913 10.1161/CIR.0000000000001209 (2024). 10.1161/CIR.0000000000001209 PMC12146881 38264914

[2] Günster, C., Jeschke, E., Malzahn, J. & Schillinger, G. In *Qualität in Der Medizin Dynamisch Denken: Versorgung - Forschung - Markt*. 111–129 (eds Kray, R. et al.) (Springer Fachmedien Wiesbaden, 2013).

[3] Heller, G. Measurement of medical outcome quality using administative data in Germany. *Bundesgesundheitsblatt Gesundheitsforschung Gesundheitsschutz*. **51**, 1173–1182. 10.1007/s00103-008-0652-0 (2008). 18985411 10.1007/s00103-008-0652-0

[4] Maier, B. et al. Comparing routine administrative data with registry data for assessing quality of hospital care in patients with myocardial infarction using deterministic record linkage. *BMC Health Serv. Res.* **16**, 605. 10.1186/s12913-016-1840-5 (2016). 27769288 10.1186/s12913-016-1840-5 PMC5073420

[5] Iezzoni, L. I. *Risk Adjustment for Measuring Health Care Outcomes* 3rd edn (Health Administration Press, 2003).

[6] Katzan, I. L. et al. Risk adjustment of ischemic stroke outcomes for comparing hospital performance. *Stroke* **45**, 918–944. 10.1161/01.str.0000441948.35804.77 (2014). 24457296 10.1161/01.str.0000441948.35804.77

[7] McNamara, R. L. et al. Development of a hospital outcome measure intended for use with electronic health records: 30-day risk-standardized mortality after acute myocardial infarction. *Med. Care*. **53**, 818–826. 10.1097/mlr.0000000000000402 (2015). 26225445 10.1097/MLR.0000000000000402

[8] Heuschmann, P. U. et al. Development and implementation of evidence-based indicators for measuring quality of acute stroke care: the Quality Indicator Board of the German Stroke Registers Study Group (ADSR). *Stroke* **37**, 2573–2578. 10.1161/01.STR.0000241086.92084.c0 (2006). 16960092 10.1161/01.STR.0000241086.92084.c0

[9] Elixhauser, A., Steiner, C., Harris, D. R. & Coffey, R. M. Comorbidity measures for use with administrative data. *Med. Care*. **36**, 8–27. 10.1097/00005650-199801000-00004 (1998). 9431328 10.1097/00005650-199801000-00004

[10] Quan, H. et al. Coding algorithms for defining comorbidities in ICD-9-CM and ICD-10 administrative data. *Med. Care*. **43**, 1130–1139. 10.1097/01.mlr.0000182534.19832.83 (2005). 16224307 10.1097/01.mlr.0000182534.19832.83

[11] Smith, E. E. et al. Risk score for in-hospital ischemic stroke mortality derived and validated within the Get With the Guidelines-Stroke Program. *Circulation* **122**, 1496–1504. 10.1161/circulationaha.109.932822 (2010). 20876438 10.1161/CIRCULATIONAHA.109.932822

[12] Fonarow, G. C. et al. Comparison of 30-day mortality models for profiling hospital performance in acute ischemic stroke with vs without adjustment for stroke severity. *Jama* **308**, 257–264. 10.1001/jama.2012.7870 (2012). 22797643 10.1001/jama.2012.7870

[13] Wang, Y., Lim, L. L., Heller, R. F., Fisher, J. & Levi, C. R. A prediction model of 1-year mortality for acute ischemic stroke patients. *Arch. Phys. Med. Rehabil*. **84**, 1006–1011. 10.1016/s0003-9993(03)00032-7 (2003). 12881825 10.1016/s0003-9993(03)00032-7

[14] Saposnik, G. et al. IScore: a risk score to predict death early after hospitalization for an acute ischemic stroke. *Circulation* **123**, 739–749. 10.1161/circulationaha.110.983353 (2011). 21300951 10.1161/CIRCULATIONAHA.110.983353

[15] Chen, S. D. et al. Machine learning is an effective method to predict the 90-day prognosis of patients with transient ischemic attack and minor stroke. *BMC Med. Res. Methodol.* **22**, 195. 10.1186/s12874-022-01672-z (2022). 35842606 10.1186/s12874-022-01672-z PMC9287991

[16] Schwartz, J. et al. Incorporating stroke severity into hospital measures of 30-Day mortality after ischemic stroke hospitalization. *Stroke* **48**, 3101–3107. 10.1161/strokeaha.117.017960 (2017). 28954922 10.1161/STROKEAHA.117.017960

[17] Geraedts, M. et al. Quality assurance measures and mortality after stroke. *Dtsch. Arztebl Int.* **118**, 857–863. 10.3238/arztebl.m2021.0339 (2021). 34730084 10.3238/arztebl.m2021.0339 PMC8948340

[18] Nacimiento, W., Töpper, R., Erbguth, F. & Höfling, W. Schlaganfallmedizin: Mortalitätsrate Allein Kein kriterium für eine Gute versorgung. *Deutsches Ärzteblatt*. **113**, 595 (2016).

[19] Brott, T. et al. Measurements of acute cerebral infarction: a clinical examination scale. *Stroke* **20**, 864–870. 10.1161/01.str.20.7.864 (1989). 2749846 10.1161/01.str.20.7.864

[20] Adams, H. P. Jr. et al. Baseline NIH stroke scale score strongly predicts outcome after stroke: A report of the Trial of Org 10172 in Acute Stroke Treatment (TOAST). *Neurology* **53**, 126–131. 10.1212/wnl.53.1.126 (1999). 10408548 10.1212/wnl.53.1.126

[21] Barlinn, J. et al. Telemedicine in stroke-pertinent to stroke care in Germany. *Nervenarzt* **92**, 593–601. 10.1007/s00115-021-01137-6 (2021). 34046722 10.1007/s00115-021-01137-6 PMC8184549

[22] Voss, E. A. et al. Feasibility and utility of applications of the common data model to multiple, disparate observational health databases. *J. Am. Med. Inf. Assoc.* **22**, 553–564. 10.1093/jamia/ocu023 (2015). 10.1093/jamia/ocu023 PMC4457111 25670757

[23] Henke, E. et al. German claims data for real-world research: content coverage evaluation in OMOP CDM. *Stud. Health Technol. Inf.* **302**, 3–7. 10.3233/shti230053 (2023). 10.3233/SHTI230053 37203598

[24] Spoden, M. et al. A high hospital frailty risk score indicates an increased risk for complications following surgical treatment of proximal humerus fractures. *Arch. Gerontol. Geriatr.* **128**, 105598. 10.1016/j.archger.2024.105598 (2025). 39182348 10.1016/j.archger.2024.105598

[25] Team, R. C. *R: A Language and Environment for Statistical Computing* https://www.R-project.org/ (2023).

[26] Kuhn, M. Building predictive models in R using the caret package. *J. Stat. Softw.* **28**, 1–26. 10.18637/jss.v028.i05 (2008).27774042

[27] Chen, T., Guestrin, C. & XGBoost: A scalable tree boosting system. *CoRR* abs/1603.02754 (2016).

[28] Huang, J. et al. Interpretable machine learning for predicting 28-day all-cause in-hospital mortality for hypertensive ischemic or hemorrhagic stroke patients in the ICU: a multi-center retrospective cohort study with internal and external cross-validation. *Front. Neurol.* **14**, 1185447. 10.3389/fneur.2023.1185447 (2023). 37614971 10.3389/fneur.2023.1185447 PMC10443100

[29] Xu, Y. et al. Extreme gradient boosting model has a better performance in predicting the risk of 90-day readmissions in patients with ischaemic stroke. *J. Stroke Cerebrovasc. Dis.* **28**, 104441. 10.1016/j.jstrokecerebrovasdis.2019.104441 (2019). 31627995 10.1016/j.jstrokecerebrovasdis.2019.104441

[30] ems: Epimed Solutions Collection for Data Editing, Analysis, and Benchmark of Health Units v. R package version 1.3.11. (2021).

