# Supplementary material for: Evaluation of hybrid stroke quality indicators by integrating NIHSS and claims data for improved outcome prediction
Source: Sci Rep. 2025 Nov 7;15:38994. doi: 10.1038/s41598-025-25979-1 (PMC12594769; doi:10.1038/s41598-025-25979-1)
Supplement: Supplementary file 1 — Supplementary Material 1 [file 41598_2025_25979_MOESM1_ESM.pdf]

Supplement

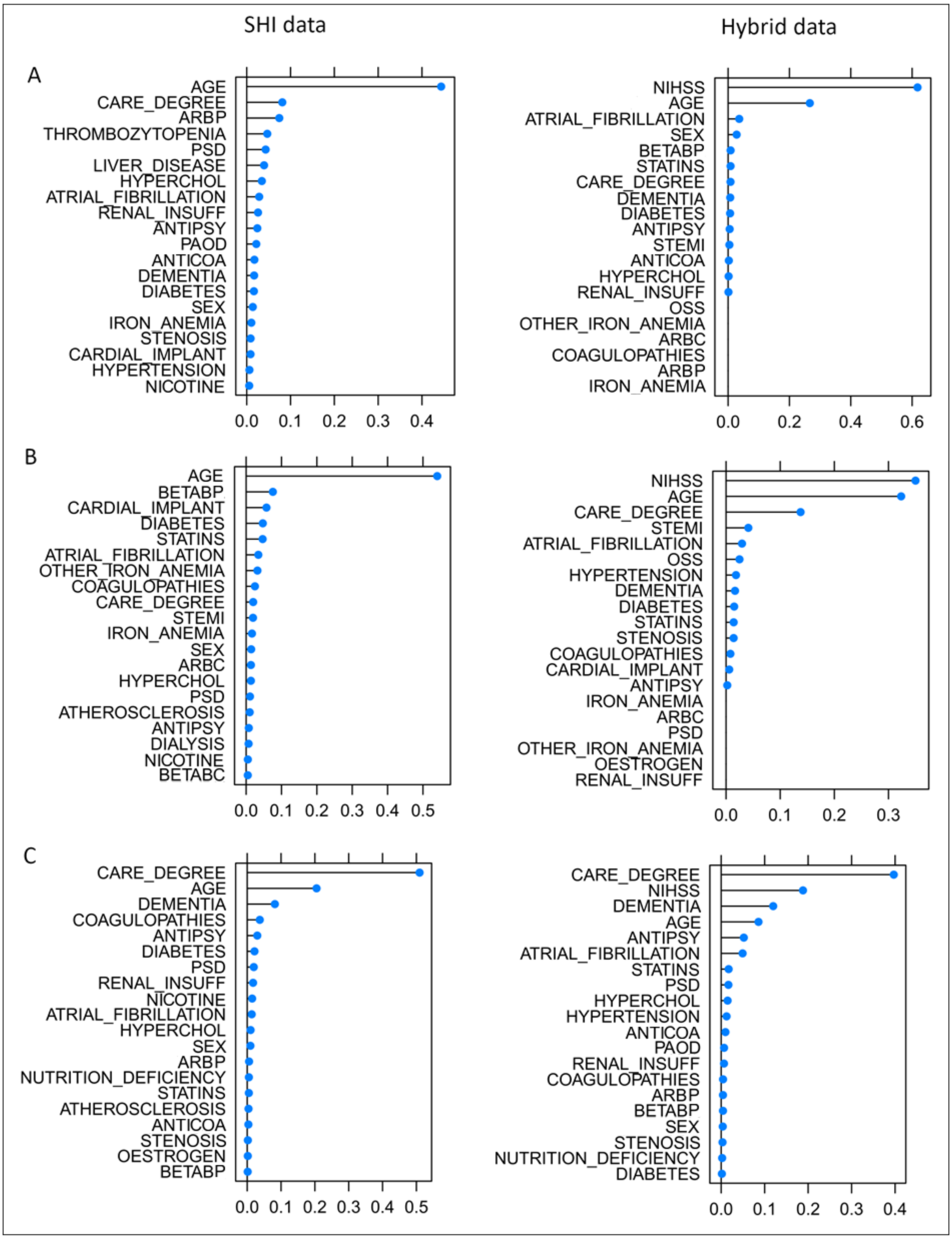

Figure S1: Comparison of variable importance (XGBoost model) between claims data (left side) and hybrid data (right side) for I61. Outcomes: A: *30-day mortality*; B: *reinfarction within 90 days*; C: *care degree increase within 180 days*; the influence of the clinical paramter NIHSS can be seen in the hybrid dataset. The influence of the NIHSS was particularly strong for the

outcomes A&B. Explanations of the abbreviated names of the predictor variables can be found in Table~1.

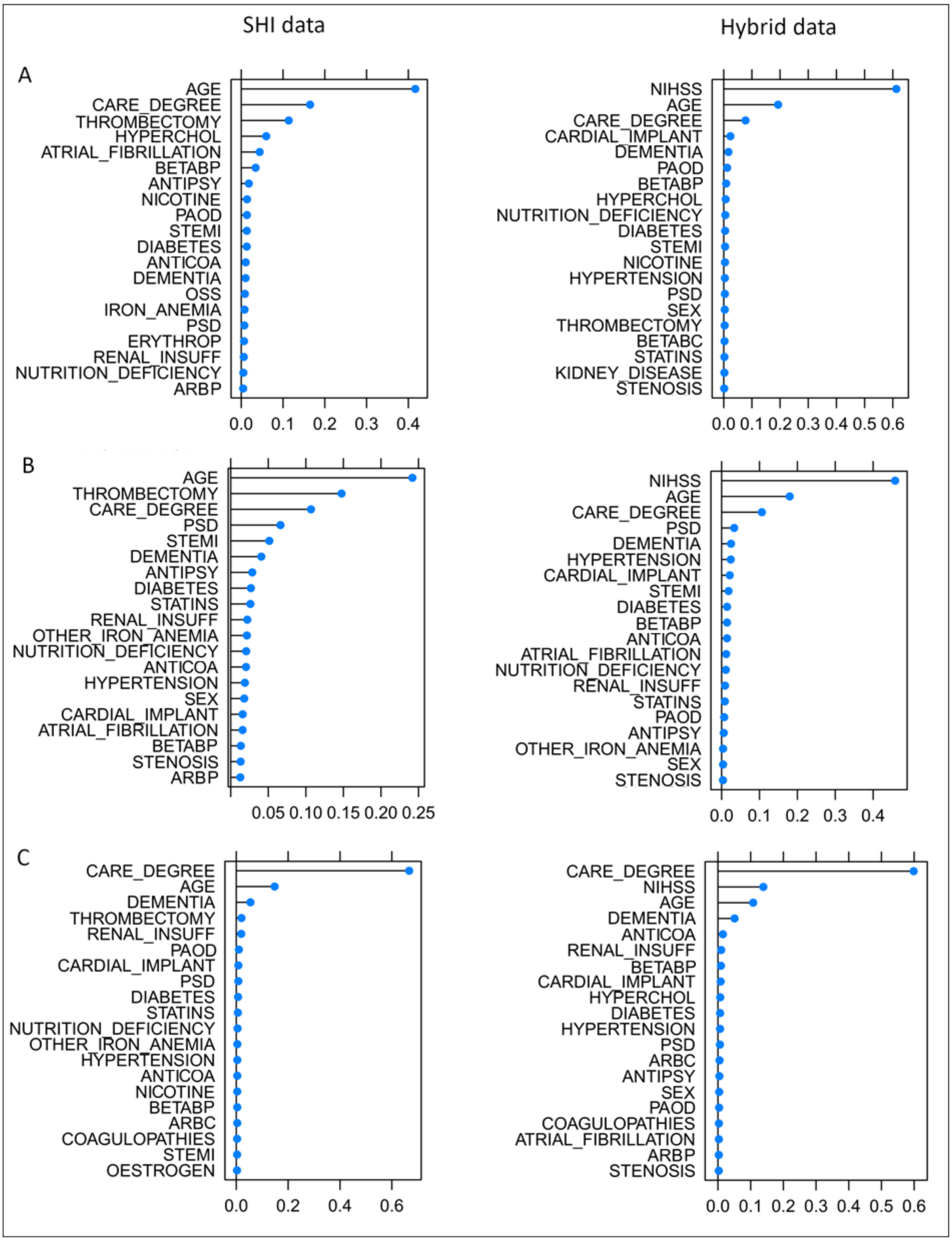

Figure S2: Comparison of variable importance (XGBoost model) between claims data (left side) and hybrid data (right side) for I63. Outcomes: A: *30-day mortality*; B: *reinfarction within 90 days*; C: *care degree increase within 180 days*; the influence of the clinical paramter NIHSS can

be seen in hybrid dataset. The influence of NIHSS was particularly strong for the outcomes A&B. Explanations of the abbreviated names of the predictor variables can be found in Table~1.

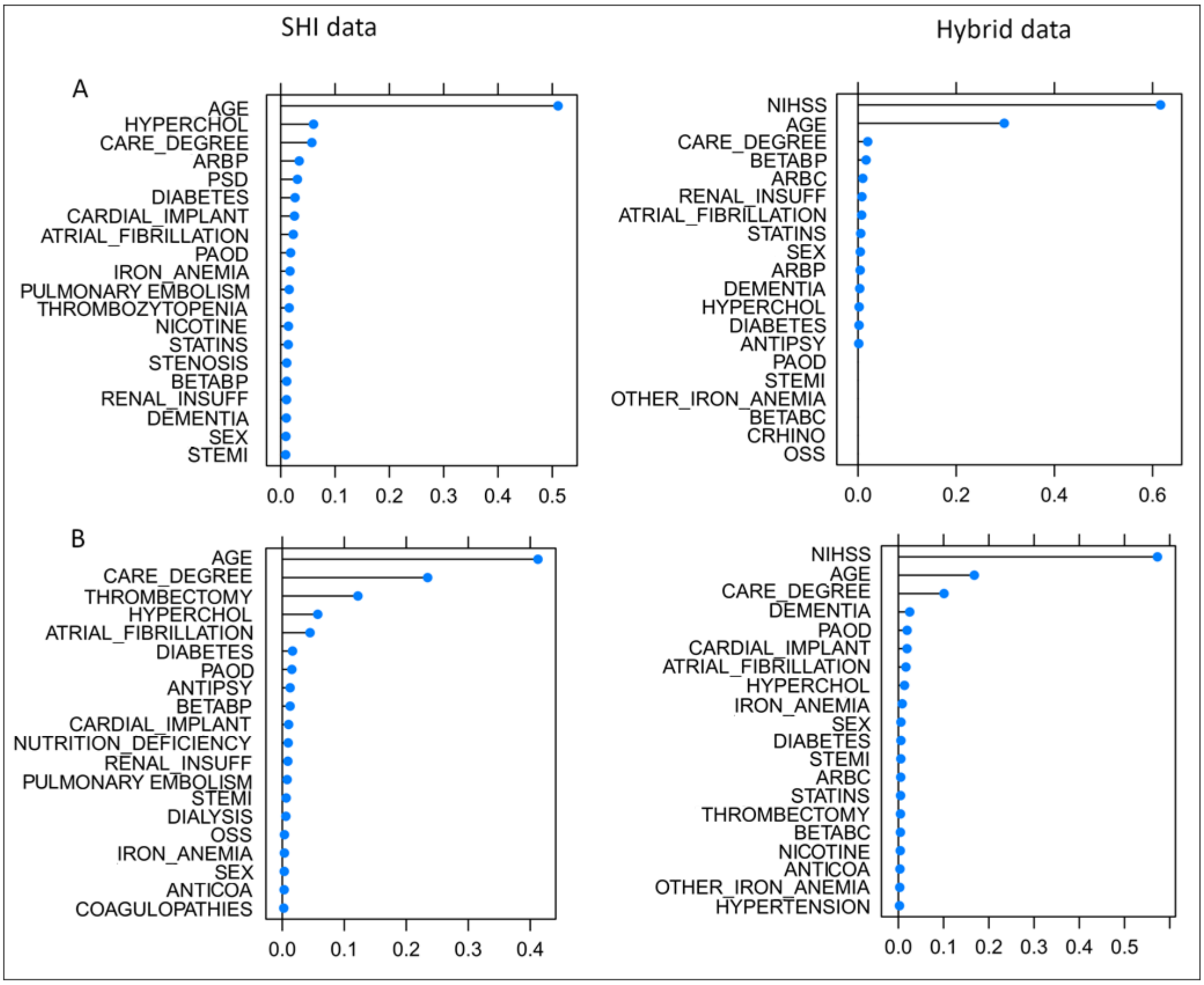

Figure S3: Sensitivity analysis comparing variable importance (XGBoost model) between claims data (left side) and hybrid data (right side) for I61 (A) and I63 (B). Outcome: *30-day mortality*; the influence of the clinical paramter NIHSS still remains in hybrid dataset after exclusion of patients with a NIHSS score of 32. Explanations of the abbreviated names of the predictor variables can be found in Table~1.

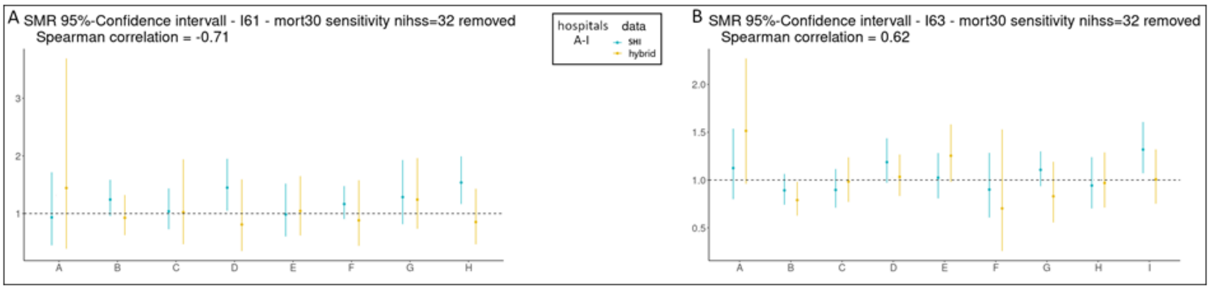

Figure S4: Sensitivity analysis comparing hospital ranking based on SMR (XGBoost model) between claims data and hybrid data. Patients with an NIHSS score of 32 were excluded. The *30-day mortality* was used as the outcome. A: Data for I61; B: Data for I63; SMR (Standardized Mortality Ratio) with 95% confidence interval and Spearman correlation between claims data and hybrid data are given for each comparison.

Table S1: Claims data based risk factors used for modeling

	Entity	
	**I61**	**I63**
Risk factors	Sex, Age, Dementia, Hypercholesterolaemia, Nicotine abuse, Obstructive sleep apnoea syndrome (OSS), Peripheral arterial occlusive disease (POAD), Post stroke depression (PSD), Chronic rhinositis, STEMI, Stenosis (cerebral atherosclerosis), Atherosclerosis, Pulmonary embolism, Iron deficiency anaemia after blood loss (chronic), other iron deficiency anaemias, Dialysis, Cardial implantation, Diabetes (E10-E14), Thrombocytopenia, Anaemia, Renal insufficiency, Hypertension, Atrial fibrillation and flutter, Liver disease, Nutritional deficiency, Coagulopathies, Care degree, medications: anticoagulants, statins, angiotensin receptor blocker pure/combined, beta blocker pure/combined,	Sex, Age, Dementia, Hypercholesterolaemia, Nicotine abuse, Obstructive sleep apnoea syndrome (OSS), Endocarditis, Vasculopathies, Hypertensive kidney disease with renal insufficiency, Erythrocytopenia, Peripheral arterial occlusive disease (POAD), Post stroke depression (PSD), Chronic rhinositis, STEMI, Stenosis (cerebral atherosclerosis), Pulmonary embolism, Iron deficiency anaemia after blood loss (chronic), other iron deficiency anaemias, Dialysis, Cardial implantation, Diabetes (E10-E14), Thrombocytopenia, Anaemia, Renal insufficiency, Hypertension, Atrial fibrillation and flutter, Liver disease, Nutritional deficiency, Coagulopathies, Care degree, Thrombectomy during index stay, medications: anticoagulants,

	antipsychotics, estrogen therapy	statins, angiotensin receptor blocker pure/combined, beta blocker pure/combined, antipsychotics, estrogen therapy
